# Interaction of peroxiredoxin V with dihydrolipoamide branched chain transacylase E2 (DBT) in mouse kidney under hypoxia

**DOI:** 10.1186/s12953-014-0061-2

**Published:** 2015-02-05

**Authors:** Sun Hee Ahn, Hee-Young Yang, Gia Buu Tran, Joseph Kwon, Kyu-Yeol Son, Suhee Kim, Quoc Thuong Dinh, Seunggon Jung, Ha-Mi Lee, Kyoung-Oh Cho, Tae-Hoon Lee

**Affiliations:** Department of Oral Biochemistry, Dental Science Research Institute, Medical Research Center for Biomineralization Disorders, School of Dentistry, Chonnam National University, 300 Yongbong-Dong, Buk-Ku, Gwangju, 500-757 Republic of Korea; Department of Molecular Medicine, Graduate School, Chonnam National University, Gwangju, Republic of Korea; Korea Basic Science Institute, Daejeon, Republic of Korea; Laboratory of Veterinary Pathology, College of Veterinary Medicine, Chonnam National University, Gwangju, Republic of Korea; Department of Oral and Maxillofacial Surgery, School of Dentistry, Chonnam National University, Gwangju, Republic of Korea

**Keywords:** Peroxiredoxin V, Hypoxia, Kidney, Dihydrolipoamide branched-chain transacylase, Peroxidatic cysteine

## Abstract

**Background:**

Peroxiredoxin V (Prdx V) plays a major role in preventing oxidative damage as an effective antioxidant protein within a variety of cells through peroxidase activity. However, the function of Prdx V is not limited to peroxidase enzymatic activity per se. It appears to have unique function in regulating cellular response to external stimuli by directing interaction with signaling protein. In this study, we identified Prdx V interacting partners in mouse kidney under hypoxic stress using immunoprecipitation and shotgun proteomic analysis (LC-MS/MS).

**Results:**

Immunoprecipitation coupled with nano-UPLC-MS^E^ shotgun proteomics was employed to identify putative interacting partners of Prdx V in mouse kidney in the setting of hypoxia. A total of 17 proteins were identified as potential interacting partners of Prdx V by a comparative interactomics analysis in kidney under normoxia versus hypoxia. Dihydrolipoamide branched chain transacylase E2 (DBT) appeared to be a prominent candidate protein displaying enhanced interaction with Prdx V under hypoxic stress. Moreover, hypoxic kidney exhibited altered DBT enzymatic activity compared to normoxia. An enhanced colocalization of these two proteins under hypoxic stress was successfully observed in vitro. Furthermore, peroxidatic cysteine residue (Cys48) of Prdx V is likely to be responsible for interacting with DBT.

**Conclusions:**

We identified several proteins interacting with Prdx V under hypoxic condition known to induce renal oxidative stress. In hypoxic condition, we observed an enhanced interaction of Prdx V and DBT protein as well as increased DBT enzymatic activity. The results from this study will contribute to enhance our understanding of Prdx V’s role in hypoxic stress and may suggest new directions for future research.

**Electronic supplementary material:**

The online version of this article (doi:10.1186/s12953-014-0061-2) contains supplementary material, which is available to authorized users.

## Background

Peroxiredoxins (Prdxs) are known to include six isoforms with different cellular localizations, and constitute 0.1–1% of total soluble protein in most human cells [1]. Prdxs play major roles in preventing oxidative damage as the effective antioxidant proteins within a variety of cells through peroxidase activity [2]. In mammalian cells, Prdx V was identified originally as a transcriptional corepressor [3], and a peroxisomal and mitochondrial antioxidant protein [4]. Recently, Prdx V was found to be a stress-inducible factor under specialized oxidative stress conditions, especially hypoxic stress [5]. This protein appears to be multifunctional, and the full spectrum of cellular functions of Prdx V remains unknown.

Hypoxia is one of the most important factors influencing clinical outcomes in the renal environment [6]. A growing body of literature has implicated hypoxia in the pathogenesis of both acute and chronic renal disease [7-9]. The partial pressure of oxygen (pO_2_) in the kidney is ordinarily sustained at well-balanced levels by complex functional interactions among renal blood flow, glomerular filtration rate, O_2_ consumption, and arteriovenous O_2_ shunting [10]. Kidney is particularly susceptible to hypoxic damage depending on the delicacy of these complex functional interactions. Renal cells use various molecular pathways that allow them to respond and adapt to changes in renal oxygenation [11]. The efficient metabolic acclimation to low pO_2_ is thus a vital factor for maintenance of renal transport ability and essential for cell survival. For example, the prolyl-4-hydroxylase domain (PHD)/hypoxia-inducible factor (HIF) pathway has a primal role in metabolic reprogramming under low pO_2_ conditions so that it regulates cellular energy and glucose metabolism at multiple levels. HIFs alter metabolic conditions from oxidative phosphorylation to anaerobic glycolysis, and inhibit mitochondrial respiration and ROS generation. It does this by increasing the expression of glycolytic enzymes, by blocking the conversion of pyruvate to acetyl CoA through transcriptional upregulation of pyruvate dehydrogenase kinase, and by regulating the expression of proteins that compose the mitochondrial respiratory chain [11]. Also, mitochondrial biogenesis and its consequent processes enhance metabolic pathways such as fatty acid oxidation and boost antioxidant defense mechanisms that remediate injury from tissue hypoxia, and glucose or fatty acid overburden, all of which would otherwise contribute to the pathogenesis of acute and chronic kidney disease [12].

Our previous study indicated that Prdx V exerted protective effects in the hypoxic kidney by regulating a variety of individual proteins in a protein network. Using shotgun proteomic analysis, it has been shown that knocking down Prdx V influences the expression of a variety of protein groups associated with oxidative stress, mitochondrial transport, fatty acid metabolism, amino acid/nucleic acid metabolism, glycolysis/gluconeogenesis, and the cytoskeleton. Additionally, the hypoxic kidneys in Prdx V knock-down mice (Prdx V^si^) showed insufficient activities of mitochondrial metabolic enzymes, especially aconitase 2 (Aco2), acyl-CoA dehydrogenase C-4 to C-12 straight chain (Acadm), and acyl-CoA oxidase 1 (Acox1) [13]. Taken together, these findings suggest that Prdx V may be involved in the coupling of a broad range of cellular signaling cascades to maintain renal homeostasis under hypoxic conditions. To gain further insights into the mechanisms regulated by Prdx V in hypoxic conditions, we employed an approach to compare the interacting partners in the kidneys under normoxia versus hypoxia. Here, we report Prdx V interactome using a strategy of immunoprecipitating Prdx V-protein complexes in the hypoxic kidney. Our data reveal a promising target protein interacting with Prdx V in the hypoxic kidney and provide a potential therapeutic target for chronic kidney disease.

## Results

### Identification of Prdx V interactome in normoxic and hypoxic mouse kidney

Previously, we reported that Prdx V was a novel regulator of renal homeostasis under hypoxic stress, altering protein networks associated with oxidative stress, fatty acid metabolism, and mitochondrial dysfunction [13]. However, it is still unclear which protein/molecules interact with Prdx V in regulating kidney homeostasis. To capture potential interaction partners of mouse Prdx V, we used an anti-Prdx V antibody to immunoprecipitate Prdx V and its interacting proteins from normoxic and hypoxic kidneys. The immunoprecipitates were then subjected to gel-assisted digestion followed by nano-UPLC-MS analysis. According to Han et al., to maximize protein digestion efficiency and recovery (>90%) [14], we employed the gel-assisted digestion method. We next subjected the digested protein to a nano-UPLC-MS^E^ proteomic analysis to identify proteins interacting with Prdx V. We compared the proteomic data from three independent experiments to determine meaningful targets with high reproducibility. In detail, 27 (149 spectra) and 33 (276 spectra) proteins were identified as Prdx V’s interaction proteins under normoxic and hypoxic conditions, respectively. Table 1 summarizes the potential interacting partners of Prdx V, identified under normoxic and hypoxic conditions. Among them, 13 proteins showed increased interaction with Prdx V in the hypoxic versus the normoxic kidney: Rab43, DBT, Alb, Pcca, Krt76, Krt14, Krt17, Krt84, Krt72, Krt74, Krt77, Krt42, and Pccb. On the other hand, four proteins showed decreased interaction with Prdx V in the hypoxic versus the normoxic kidney: Gba2, Txn1, Krt78, and Krt32 (Table 1).

**Table 1:** **Protein showing altered interaction by Prdx V immunoprecipitation during hypoxic stress** ^**a**^

**Accession**	**Description**	**Gene**	**Score**	**MS/MS spectra**	**Frequency** ^**b**^	
					**Normoxia**	**Hypoxia**
IPI00130467	Ras related protein Rab 43 isoform b	Rab43	228.7	6	ND	1/3
IPI00130535	Lipoamide acyltransferase component of branched chain α-keto acid dehydrogenase complex, mitochondial	Dbt	302.6	8	ND	2/3
IPI00131695	Serum albumin	Alb	325.3	3	1/3	3/3
IPI00225123	Non-lysosomal glucosylceramidase	Gba2	191.4	9	1/3	ND
IPI00226993	Thioredoxin	Txn1	1155.1	3	3/3	2/3
IPI00330523	Propionyl CoA carboxylase alpha chain, mitochondrial	Pcca	474.0	21	2/3	3/3
IPI00346834	Keratin type II cytoskeletal 2, oral	Krt76	184.2	7	ND	1/3
IPI00348328	Keratin Kb40	Krt78	580.5	19	1/3	ND
IPI00122281	Keratin type I cuticular Ha2	Krt32	260.3	8	1/3	ND
IPI00227140	Keratin type I cytoskeletal 14	Krt14	1399.7	16	ND	1/3
IPI00230365	Keratin type I cytoskeletal 17	Krt17	1322.1	16	ND	1/3
IPI00347019	Keratin type II cuticular Hb4	Krt84	330.8	10	ND	1/3
IPI00347096	Keratin type II cytoskeletal 72	Krt72	337.2	8	ND	1/3
IPI00420970	Keratin type II cytoskeletal 74	Krt74	1999.7	4	1/3	3/3
IPI00462140	Keratin type II cytoskeletal 1b	Krt77	2014.2	7	ND	1/3
IPI00468696	Keratin type I cytoskeletal 42	Krt42	798.6	12	ND	1/3
IPI00606510	Propionyl CoA carboxylase beta chain, mitochondrial	Pccb	266.6	10	2/3	3/3

^a^Proteins were affinity-purified from mouse kidneys under both normoxic and hypoxic conditions as bound interactors with Prdx V immunoprecipitation. The purified immunoprecipitates were applied to acrylamide gel-associated tryptic digestion and subjected to nano-UPLC-MS/MS for protein identification.

^b^Frequency represents the number of times that the interactors were observed in three independent experiments. ND, not detected.

To confirm our proteomics analysis for identifying Prdx V interaction partners, coprecipitation experiments were performed, targeting some representative proteins. As shown in Additional file 1: Figure S1, Rab43, Alb, and Pccb were shown to strongly coprecipitate with Prdx V in hypoxia, consistent with the proteomics analysis in Table 1. Taken together, these findings suggest that Prdx V could act as a direct regulator in hypoxia and be involved in maintaining kidney homeostasis.

### Interaction of Prdx V and DBT in hypoxic mouse kidney

**Figure 1:**
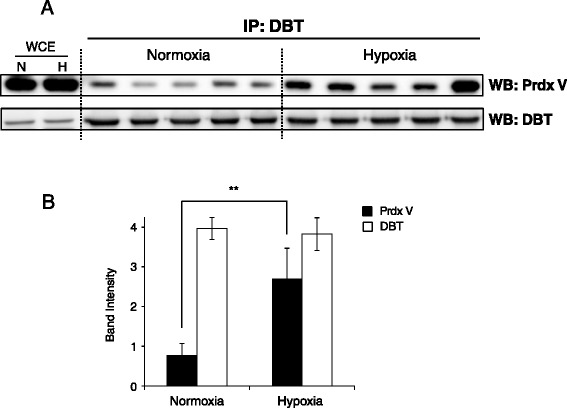
**Coprecipitation of endogenous Prdx V and DBT in normoxic and hypoxic mouse kidney. (A)** Immunoprecipitates identified by DBT from normoxic and hypoxic (H, lanes 3, 4) mouse kidney were probed with a Prdx V antibody (n = 5). **(B)** Histogram showing numerical data obtained by densitometry analysis of **(A)**. The histogram graph is expressed as mean values ± standard deviation (**p < 0.05). WCE, whole cell lysate of mouse kidney.

Of the Prdx V-interacting proteins, DBT appeared to be an interesting target. As shown in Table 1, DBT was identified two of three times in hypoxia, whereas it was not detected in normoxia. In our previous study, DBT protein appeared to be associated with Prdx V in hypoxic stress [13]. DBT is known as a transacylase component of the mitochondrial multienzyme BCKDH complex and it regulates branched-chain amino acid (BCCA) degradation. This raises the questions as to whether hypoxia enhances the Prdx V-DBT interaction and/or influences DBT enzymatic activity. Thus, we first confirmed the protein interaction using a reverse immunoprecipitation (Figure 1A). As a result, we found a four-fold higher interaction between Prdx V and DBT in hypoxia than normoxia (Figure 1B). To our knowledge, this is the first report showing that Prdx V could bind directly to DBT and this interaction was enhanced by hypoxic stress, which might suggest a strong linkage to amino acid metabolism and oxidative stress.

**Figure 2:**
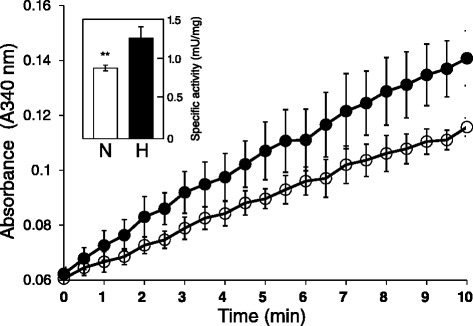
**In vitro activities of DBT enzyme in normoxic and hypoxic mouse kidneys.** Plots show time-dependent changes in absorbance at 340 nm. The histograms in the insets show the slopes (rate) of the changes in the linear portion of the curves. The y-axes in the histograms indicate the specific activity expressed as mU/mg mitochondrial protein. 1 mU is defined as the amount of enzyme needed to reduce 1 nmol of NAD^+^ per min at 37°C. Assays were carried out with the mitochondrial fraction of homogenized tissue extract, and the reactions were run using sodium α-ketoisocaproate as the substrate. Data are expressed as mean values ± standard deviation (**p < 0.05). Open circles and histogram, normoxia; closed circles and histogram, hypoxia.

To examine the effect of hypoxia on DBT enzymatic activity, we assayed DBT enzymatic activities in mouse kidneys under normoxic and hypoxic conditions. DBT enzymatic activities in hypoxic and normoxic mouse kidneys were 1.20 ± 0.05 and 0.88 ± 0.04 mU/mg, respectively (n = 10 mice for each group; Figure 2). This indicates that hypoxic treatment resulted in a significant increase in DBT activity (about 1.5-fold) versus normoxic conditions (Figure 2, inset bar graph).

**Figure 3:**
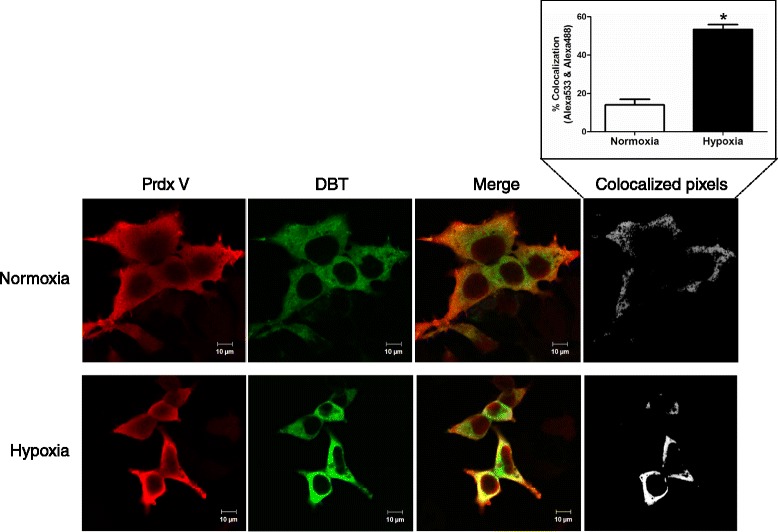
**Co-localization of Prdx V and DBT in normoxic and hypoxic HEK293 cells.** Confocal images of costaining with antibodies to Prdx V and DBT. HEK293 cells were co-transfected with Prdx V- and DBT-expressing vectors, and then the transfected cells were exposed to hypoxic stress (1 ± 0.2%) for 6 h. Cells were fixed on polylysine slides and stained with Prdx V (red) or DBT (green) antibodies.

We next performed co-immunostaining of Prdx V and DBT to determine their subcellular co-localization in normoxic and hypoxic conditions. HEK293 cells were cotransfected with a myc-tagged DBT and a HA-tagged Prdx V. As shown in Figure 3, DBT (green) was colocalized with Prdx V proteins (red) in hypoxic HEK293 cells, while the complex was weakly detectable in normoxic cell. Colocalized pixels were quantified as the percentage of overlapping selected red (Prdx V) and green pixels (DBT) and revealed that co-localization of these proteins increased 3.8-fold in hypoxia versus normoxia (Figure 3, inset bar graph).

### Cysteine 48 of Prdx V is required for the interaction with DBT

**Figure 4:**
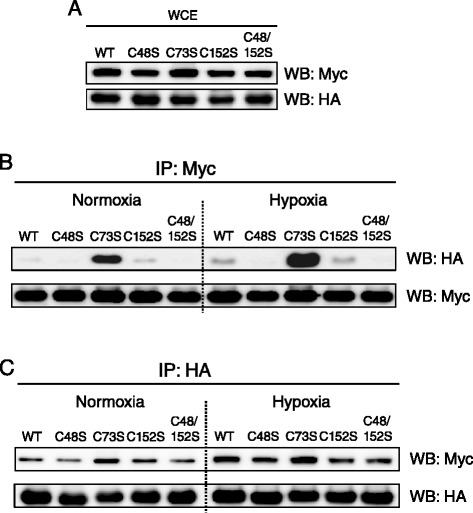
**Comparative interactions of Prdx V WT or cysteine mutants with DBT in normoxic and hypoxic cells. (A)** All WCE presented for every Prdx V mutant showed the same background of cellular expression for each transfected construct. **(B)** Coprecipitation of HA-tagged Prdx V with myc-tagged DBT. **(C)** Reverse immunoprecipitation of myc-tagged DBT with HA-tagged Prdx V. HEK293 cells were cotransfected with the HA-tagged Prdx V WT or cysteine mutant (C48S, C73S, C152S, C48/152S) and myc-tagged DBT expression vector, and then incubated under normoxic and hypoxic conditions. WCE, whole cell lysate of HEK293 cell; Mock, the empty vector tagged to myc or HA.

Human Prdx V has one catalytic cysteine residue, Cys 48, which is highly conserved in all other peroxiredoxins (Prdx I-IV and VI), and this cysteine residue is involved in Prdx V’s peroxidase activity [15]. Most recently, we demonstrated that Prdx V interacts with Jak2 via the catalytic Cys 48 to modulate the Jak2-Stat5 pathway [16]. Based on our previous observations, we next asked whether the catalytic Cys 48 plays a role in the Prdx V-DBT interaction. To test this, we transiently co-transfected HEK293 cells with a myc-tagged DBT and a HA-tagged wild-type (WT) or a cysteine mutant Prdx V (C48S, C73S, C152S, and C48/152S) expression construct. Then, total cell lysate proteins from transfected-HEK293 cells under normoxia and hypoxia were used for immunoprecipitations with anti-myc-conjugated agarose. The cellular expression of DBT and PrdxV for each transfected construct showed the same background for every Prdx V mutant (Figure 4A). As shown in Figure 4B, WT Prdx V bound weakly to DBT under normoxic conditions, but this interaction showed a significant increase under hypoxic stress. In the case of the Prdx V C152S mutant, the interaction with DBT did not change compared with WT Prdx V. However, Prdx V C48S mutant and double mutant (C48/152S) showed diminished interactions with DBT under both normoxic and hypoxic conditions. This result is consistent with our previous finding of the cysteine 48 residue’s role in the interaction of Prdx V and Jak2. Additionally, Prdx V C73S showed a notable increase in the interaction with DBT in both normoxic and hypoxic conditions. This needs further investigation. Using reverse immunoprecipitation, we also found that Prdx V and DBT interaction was increased in hypoxia. Although Prdx V 73S showed increased interaction with DBT, Prdx V C48S showed decreased interaction with DBT (Figure 4C). Thus, these data indicate that the Prdx V and DBT interaction during normoxic and hypoxic conditions requires the catalytic cysteine of Prdx V.

## Discussion

Prdx V has a remarkably wide subcellular distribution compared with the other mammalian peroxiredoxins [17]. Although Prdx V appears to be constitutively and ubiquitously expressed in most mammalian tissues, its expression is also upregulated in various pathophysiological situations in response to various stresses [15].

In this study, we employed a manageable and rapid protocol by combining Prdx V immunoprecipitation and shotgun proteomics (LC-MS/MS) to identify Prdx V and its interacting proteins in the hypoxic mouse kidney. Using this approach, we found several novel partners interacting with Prdx V from three independent replicates (Table 1). To confirm our proteomics analyses, co-immunoprecipitations were conducted using anti-target antibodies in normoxic and hypoxic mouse kidneys, and our candidate targets were validated (data not shown). Using functional annotation analysis, some Prdx V-interacting partners, such as DBT, Pcca, and Pccb, appeared to be related to various mitochondria-associated metabolism functions. This is consistent with a previous report regarding the targets in Prdx V knock-down mouse kidney under hypoxia [13]. Of note, Prdx V knockdown might cause an imbalance in the kidney under hypoxic stress by altering metabolism and causing mitochondrial dysfunction.

The BCKDH complex is an inner-mitochondrial enzyme complex involved in the breakdown of BCAAs, such as isoleucine, leucine, and valine, which is thought to be composed of a core of 24 transacylase (E2) subunits, and associated with decarboxylase (E1), dehydrogenase (E3), and regulatory subunits [18,19]. BCAAs are essential amino acids that serve as substrates for protein synthesis and also function in nutritional signals, regulating carbohydrate metabolism, energy balance and hormone secretion. Thus, the homeostasis of BCCAs is tightly regulated by the BCKDH complex. In particular, DBT belongs to the transacylase (E2) subunit and is a key enzyme in amino acid metabolism. Each E2 subunit consists of three independently functional domains: a lipoyl-bearing domain located in the N-terminal portion, an E1/E3-binding domain, and an inner-core domain at the C-terminal portion, with the three domains tethered by flexible linker regions [20]. The core domains of E2 subunit form a 24-meric scaffold, which is decorated with multiple copies of E1 and E3 attached through the subunit-binding domain [19]. Although the basic enzyme activity of DBT is known, along with the protein sequence and structure, the physiological functions in amino acid metabolism under hypoxic stress are still unknown. Although BCAAs are essential amino acids, accumulation of BCAAs and their metabolites can be toxic to cells; thus, hypoxia-induced BCAA accumulation may promote kidney injury. The regulation of free amino acid levels is an important factor in understanding amino acid metabolism in acute hypoxia on tissues. Here, we identified that the reinforced interaction between Prdx V and DBT in mouse kidney was inducible by hypoxic stress. Additionally, we found that the enzyme activity of DBT was increased in hypoxia versus normoxia. Thus, we suggest that Prdx V may be an important molecule for the regulation of DBT activity through a direct interaction under hypoxic stress.

Unlike other members of Prdx family, human Prdx V does not contain a cysteine residue corresponding to the second conserved cysteine residue of the 2-Cys subgroup but possesses two additional cysteine residues (C73 and C152) that are lacking in the 1-Cys subgroup; thus, Prdx V belongs to the atypical 2-Cys family [15]. The atypical 2-Cys human Prdx have side chains for all Cys residues (the peroxidatic Cys48, the resolving Cys152, and the additional Cys73) characterized by the presence of a free sulfhydryl group [21]. The peroxidatic residue Cys48 of Prdx V is a highly conserved Cys residue in all peroxiredoxins. This residue is located within the N-terminal part of the α2-helix, twisted at Ala60, in the reduced form, and lies in the positive charged active-site pocket built by surrounding factors such as Arg128, Thr45, and Val40 [15]. The resolving residue Cys152, located in the loop between β7 and α6, is associated with the proposed enzymatic mechanism of action of Prdx V. The proposed enzymatic mechanism requires the formation of an intramolecular disulfide bond between these two Cys residues. The additional Cys, Cys73, is found in the C-terminal part of the β4 stand of human Prdx V. However, this residue is not implicated in the enzymatic mechanism, because its mutation does not modify the activity [17]. It is thus surprising to find this Cys73 located close to the peroxidatic Cys48, at the bottom of the active-site pocket. Although the enzymatic mechanism involving the cysteine residues is well established, the physiological functions under specific stimulation conditions are still unknown. Other investigators have demonstrated that the catalytic cysteine of Prdx V has an important role in cytoprotection effects via its antioxidant activity [22]. Moreover, we recently reported a novel function of the peroxidatic Cys48 as a specific regulator of JAK2-Stat5 signaling triggered by lipopolysaccharide (LPS) [16]. Notably, it appeared that Prdx V played a role in the regulation of IL-6 expression through direct binding to Jak2 via its catalytic Cys48 residue, although this did not involve its peroxidase activity. Consistent with that, our current data show that hypoxia enhanced the direct interaction between Prdx V and DBT, and this enhancement did require the catalytic cysteine residue, Cys48, of Prdx V but not the other cysteine residues (Cys73 and Cys152).

In summary, this study provides new findings of novel Prdx V-interacting partners and showed alteration of DBT enzymatic activity in the kidney under hypoxia, which may be important for the kidney pathological conditions, such as hypoxic stress. Further studies are needed to explore the exact mechanism underlying Prdx V-DBT protein interaction associated with kidney homeostasis in chronic renal disease.

## Conclusions

We have shown that Prdx V protein was interacting with other proteins in mouse kidney responding to hypoxic stress. One of interacting partner, DBT, showed enhanced interaction with Prdx V under hypoxia. Interestingly, DBT enzymatic activity, known to be involved in mitochondrial metabolism, was increased under hypoxia as well. In addition, peroxidatic cysteine (Cys48) of Prdx V appeared to be important for interaction with DBT. This study proposes that Prdx V interacts with DBT and this interaction may be associated with mitochondrial metabolism in mouse kidney under hypoxia. Further functional analysis is required to confirm this hypothesis and to elucidate the underlying the role of Prdx V in biological mechanisms of hypoxic stress-induced mitochondrial metabolism.

## Methods

### Hypoxic conditions, protein extraction and immunoprecipitation

Mice (C57BL/6 J) were maintained under specific pathogen-free (SPF) conditions. All animal-related procedures were reviewed and approved under the Animal Care Regulations (ACR) of Chonnam National University (accession number: CNU IACUC-YB-2013-39).

Hypoxic stress was produced as described previously. Briefly, mice were placed in a chamber designed to regulate the flow of N_2_ using a gas supply and the oxygen concentration was maintained at 8 ± 0.5% O_2_ using an oxygen controller (Proox Model 110; BioSpherix, NY, USA). After 4 h of hypoxia, all mice (n = 10/each group, 8 weeks old) were induced with anesthesia under hypoxic conditions, and the kidneys were rapidly collected and frozen in liquid N_2_ [13].

Total protein extraction was performed according to Yang et al. [13]. For immunoprecipitation assays, 500 μg of total extracted proteins was incubated with 10 μL of anti-Prdx V antibody at 4°C overnight. Then, immune complexes were pulled down by incubating with protein G agarose (Invitrogen, Carlsbad, CA) for 4 h at 4°C. The immunoprecipitated complex was eluted with 60 mM Tris–HCl (pH 6.8), 2.5% glycerol, 2% SDS, and 28.8 mM β-mercaptoethanol, and then the eluted complex was freeze-dried before being subjected to nano-UPLC-MS/MS analysis for comparative proteomics.

### Interactome analysis by nano-UPLC-MS/MS

For gel-assisted digestion, the dried pellet was resuspended in 50 μl of 6 M urea, 5 mM EDTA, and 2% (w/v) SDS in 0.1 M tetra ethyl ammonium bicarbonate (TEABC) and incubated at 37°C for 30 min for complete dissolution. Proteins were reduced by adding 10 μL of 20 mM tris-2-carboxyethyl phosphine (TCEP) and alkylated followed by adding 20 μl of 20 mM iodoacetamide (IAM) at room temperature for 30 min. To incorporate proteins into a gel directly in the Eppendorf vial, 18.5 μl of acrylamide/bisacrylamide solution (40%, v/v, 29:1), 2.5 μl of 10% (w/v) ammonium persulfate, and 1 μl of 100% TEMED was then applied to the protein solution. The gel was cut into small pieces and then washed three times with three volumes of TEABC containing 50% (v/v) ACN. The dehydrated gel samples were then digested with 15 μl trypsin (0.1 μg/μl) at 37°C for 18 hr. Then the digested peptides were recovered twice with a solution containing 50 mM ammonium bicarbonate, 50% acetonitrile, and 5% trifluoroacetic acid (TFA). The resulting peptide extracts were pooled, dried in a vacuum centrifuge, and then dissolved in 0.1% formic acid solution prior to MS or MS/MS analysis [14].

For nano-LC and tandem MS analysis, a nano-ACQUITY Ultra Performance LC Chromatography™ equipped Synapt™ G2-S System (Waters Corporation, MA, USA) used was previously described [23]. This step was performed on a 75 μm × 250 mm nano-ACQUITY UPLC 1.7 μm BEH300 C18 RP column and a 180 μm × 20 mm Symmetry C18 RP 5 μm enrichment column using a nano-ACQUITY Ultra Performance LC Chromatography™ System (Waters Corporation, MA, USA). Trypsinized peptides (5 μL) were loaded onto the enrichment column in mobile phase A (3% acetonitrile in water with 0.1% formic acid). A step gradient was then used at a flow rate of 300 nL/min. This included 3–40% mobile phase B (97% acetonitrile in water with 0.1% formic acid) run over 95 min, followed by 40–70% mobile phase B run over 20 min, and finally a sharp increase to 80% B over 10 min. Sodium formate (1 μmol/min) was used to calibrate the TOF analyzer in the range of m/z 50–2000, and [Glu1]-fibrinopeptide (m/z 785.8426) was run at 600 nL/min for lock mass correction. During data acquisition, the collision energies of low-energy mode (MS) and high-energy mode (MS^E^) were set to 4 eV and 15–40 eV energy ramping, respectively. One cycle of the MS and MS^E^ modes of acquisition was performed every 3.2 s. In each cycle, MS spectra were acquired for 1.5 s with a 0.1 s interscan delay (m/z 300–1990), and the MS^E^ fragmentation (m/z 50–2000) data were collected in triplicate.

The continuum LC-MS^E^ data were processed and searched using the IDENTITYE algorithm in PLGS (ProteinLynx GlobalServer) version 2.5.2 (Waters Corporation). The data acquired by alternating low and high energy modes in the LC-MS^E^ were automatically smoothed, background subtracted, centered, deisotoped and charge state reduced, after which alignment of the precursor and fragmentation data were combined with retention time tolerance (±0.05 min) using PLGS software.

Processed ions were mapped against the IPI mouse database (version 3.87) using the following parameters: peptide tolerance, 10 ppm; fragment tolerance, 0.05 Da; missed cleavage, 1; and carbamidomethylation at C and oxidation at methionine and cysteine. Peptide identification was performed using the trypsin digestion rule with one missed cleavage. As a result, protein identification was completed with arrangement of at least two peptides. All proteins identified on the basis the IDENTITYE algorithm are in keeping with > 95% probability. The false positive rate for protein identification was set at 5% in the databank search query option, based on the automatically generated reversed database in PLGS 2.5.2. Protein identification was also based on the assignment of at least two peptides comprised of seven fragments or more [23].

### Cell culture and transfection

HEK293 cells were cultured in Eagle’s Minimal Essential Medium (MEM) supplemented with 10% (v/v) fetal bovine serum (FBS), 1% glutamine, 100 mg/ml streptomycin, and 100 units/ml penicillin at 37°C in a humidified 5% CO_2_ atmosphere. Lipofectamine 2000 (Invitrogen) reagents were used for transfection according to the manufacturer’s instructions. At 48 hr after transfection, cells for hypoxic treatment were moved to a hypoxic incubator, and exposed to hypoxic stress, 1 ± 0.2% O_2,_ for 6 h.

### Plasmid construction

Mouse Prdx V expression (WT, C48S, C73S, C152S, and C48/152S) vectors with N-terminal HA-tags were constructed as in our previous study [24]. The full-length DBT cDNA was purchased from Korea Human Gene Bank (clone ID: hMU001786). The human DBT was subcloned into the pCMV-Myc-N vector with N-terminal myc-tag (Clontech Laboratories, CA, USA).

### DBT enzymatic activity

To measure the enzymatic activity of DBT, mitochondrial proteins from mouse hypoxic kidney (n = 10/each group) were isolated using the Qproteome Mitochondria Isolation Kit (Qiagen Sciences, Valencia, CA) according to the manufacturer’s instructions. Using isolated mitochondrial proteins, branched-chain α-keto acid dehydrogenase (BCKDH) complex activity was determined spectrophotometrically at 37°C by measuring the rate of NADH generation from NAD^+^ in the presence of substrate for BCKDH complex as described previously [25]. The reaction was started by adding the substrate and the change in the absorbance at 340 nm was recorded for 30 min. The BCKDH specific activity was expressed in mU/mg of mitochondrial protein: 1 mU was defined as the amount of enzyme that catalyzed the formation of NADH per min at 37°C.

### Confocal fluorescence microscopy

HEK293 cells grown on poly L-lysine-treated glass coverslips were transiently transfected with Prdx V- and DBT-expressing vectors and then subjected to hypoxic stress, as described above. After hypoxic treatment, cells were fixed with 4% paraformaldehyde, permeabilized with 0.1% Triton X-100 and stained with anti-HA and anti-myc antibodies, followed by Alexa fluor555- and Alexa fluor488-conjugated second antibodies. Then, cells were washed and mounted, and examined using a LSM 710 laser scanning confocal microscope (Carl Zeiss, Jena, Germany). Images were taken with a 63× objective at identical imaging settings.

## Additional file

Additional file 1: Figure S1. Coprecipitation of endogenous Prdx V with Pccb, Rab43, and Alb proteins. Mouse kidney lysates were extracted under normoxic (N) and hypoxic (H) conditions, as indicated. The extracted kidney proteins were purified using Rab43, Pccb, or Alb antibodies, as indicated, and probed with Prdx V antibody to detect the interaction with Prdx V.

## Abbreviations

Prdx V: Peroxiredoxin V
pO2: Pressure of oxygen
PHD: Prolyl-4-hydroxylase domain
HIF: Hypoxia-inducible factor
Prdx Vsi: Prdx V knock-down
Aco2: Aconitase 2
Acadm: Acyl-CoA dehydrogenase
Acox1: Acyl-CoA oxidase 1
BCAA: Branched-chain amino acid
WT: Wild-type
LPS: Lipopolysaccharide
SPF: Specific pathogen-free
ACR: Animal Care Regulations
TCEP: Tris-2-carboxyethyl phosphine
MEM: Minimal Essential Medium
FBS: Fetal bovine serum
BCKDH: Branched-chain α-keto acid dehydrogenase

## References

[1] Wood ZA, Schroder E, Robin Harris J, Poole LB (2003). Structure, mechanism and regulation of peroxiredoxins. Trends Biochem Sci.

[2] Chae HZ, Kang SW, Rhee SG (1999). Isoforms of mammalian peroxiredoxin that reduce peroxides in presence of thioredoxin. Methods Enzymol.

[3] Kropotov AV, Tomilin NV (1996). A human B-box-binding protein downregulated in adenovirus 5-transformed human cells. FEBS Lett.

[4] Knoops B, Clippe A, Bogard C, Arsalane K, Wattiez R, Hermans C, Duconseille E, Falmagne P, Bernard A (1999). Cloning and characterization of AOEB166, a novel mammalian antioxidant enzyme of the peroxiredoxin family. J Biol Chem.

[5] Shiota M, Izumi H, Miyamoto N, Onitsuka T, Kashiwagi E, Kidani A, Hirano G, Takahashi M, Ono M, Kuwano M (2008). Ets regulates peroxiredoxin1 and 5 expressions through their interaction with the high-mobility group protein B1. Cancer Sci.

[6] Yamashita H, Avraham S, Jiang S, London R, Van Veldhoven PP, Subramani S, Rogers RA, Avraham H (1999). Characterization of human and murine PMP20 peroxisomal proteins that exhibit antioxidant activity in vitro. J Biol Chem.

[7] Eckardt KU, Bernhardt WM, Weidemann A, Warnecke C, Rosenberger C, Wiesener MS, Willam C (2005). Role of hypoxia in the pathogenesis of renal disease. Kidney international Supplement.

[8] Eckardt KU, Rosenberger C, Jurgensen JS, Wiesener MS (2003). Role of hypoxia in the pathogenesis of renal disease. Blood Purification.

[9] Fine LG, Norman JT (2008). Chronic hypoxia as a mechanism of progression of chronic kidney diseases: from hypothesis to novel therapeutics. Kidney International.

[10] Evans RG, Gardiner BS, Smith DW, O'Connor PM (2008). Intrarenal oxygenation: unique challenges and the biophysical basis of homeostasis. Am J Physiol Renal Physiol.

[11] Haase VH (2013). Mechanisms of hypoxia responses in renal tissue. J Am Soc Nephrol.

[12] Weinberg JM (2011). Mitochondrial biogenesis in kidney disease. J Am Soc Nephrol.

[13] Yang HY, Kwon J, Cho EJ, Choi HI, Park C, Park HR, Park SH, Chung KJ, Ryoo ZY, Cho KO, Lee TH (2010). Proteomic analysis of protein expression affected by peroxiredoxin V knock-down in hypoxic kidney. J Proteome Res.

[14] Han CL, Chien CW, Chen WC, Chen YR, Wu CP, Li H, Chen YJ (2008). A multiplexed quantitative strategy for membrane proteomics: opportunities for mining therapeutic targets for autosomal dominant polycystic kidney disease. Mol Cell Proteomics.

[15] Knoops B, Goemaere J, Van der Eecken V, Declercq JP (2011). Peroxiredoxin 5: structure, mechanism, and function of the mammalian atypical 2-Cys peroxiredoxin. Antioxid Redox Signal.

[16] Choi HI, Chung KJ, Yang HY, Ren L, Sohn S, Kim PR, Kook MS, Choy HE, Lee TH (2013). Peroxiredoxin V selectively regulates IL-6 production by modulating the Jak2-Stat5 pathway. Free Radic Biol Med.

[17] Seo MS, Kang SW, Kim K, Baines IC, Lee TH, Rhee SG (2000). Identification of a new type of mammalian peroxiredoxin that forms an intramolecular disulfide as a reaction intermediate. J Biol Chem.

[18] Pettit FH, Yeaman SJ, Reed LJ (1978). Purification and characterization of branched chain alpha-keto acid dehydrogenase complex of bovine kidney. Proc Natl Acad Sci U S A.

[19] Griffin TA, Lau KS, Chuang DT (1988). Characterization and conservation of the inner E2 core domain structure of branched-chain alpha-keto acid dehydrogenase complex from bovine liver. Construction of a cDNA encoding the entire transacylase (E2b) precursor. J Biol Chem.

[20] Ono K, Hakozaki M, Suzuki T, Mori T, Hata H, Kochi H (2001). cDNA cloning of the chicken branched-chain alpha-keto acid dehydrogenase complex. Chicken-specific residues of the acyltransferase affect the overall activity and the interaction with the dehydrogenase. Eur J Biochem.

[21] Declercq JP, Evrard C, Clippe A, Stricht DV, Bernard A, Knoops B (2001). Crystal structure of human peroxiredoxin 5, a novel type of mammalian peroxiredoxin at 1.5 A resolution. J Mol Biol.

[22] Dubuisson M, Vander Stricht D, Clippe A, Etienne F, Nauser T, Kissner R, Koppenol WH, Rees JF, Knoops B (2004). Human peroxiredoxin 5 is a peroxynitrite reductase. FEBS Lett.

[23] Yang HY, Kwon J, Park HR, Kwon SO, Park YK, Kim HS, Chung YJ, Chang YJ, Choi HI, Chung KJ (2012). Comparative proteomic analysis for the insoluble fractions of colorectal cancer patients. Journal of Proteomics.

[24] Choi HI, Chung KJ, Yang HY, Ren L, Sohn S, Kim PR, Kook MS, Choy HE, Lee TH (2013). Peroxiredoxin V selectively regulates IL-6 production by modulating the Jak2-Stat5 pathway. Free Radic Biol Med.

[25] Rodriguez-Bayona B, Peragon J (1998). Stimulation of rat-liver branched-chain alpha-keto acid dehydrogenase activity by chronic metabolic acidosis. Int J Biochem Cell Biol.

