# Supplementary figures and images for: Interaction of peroxiredoxin V with dihydrolipoamide branched chain transacylase E2 (DBT) in mouse kidney under hypoxia
Source: Proteome Sci. 2015 Feb 5;13:4. doi: 10.1186/s12953-014-0061-2 (PMC4323032; doi:10.1186/s12953-014-0061-2)

**Supplementary Figure 1**

**
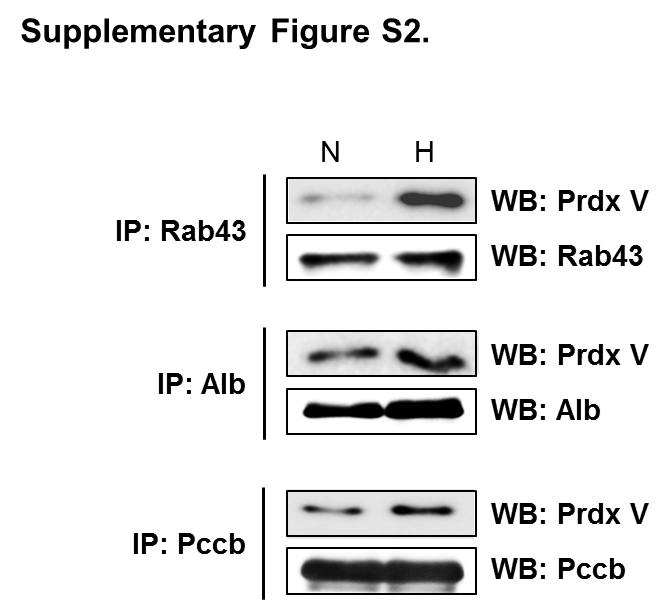
**

Supplement: Additional file 1: Figure S1. — Coprecipitation of endogenous Prdx V with Pccb, Rab43, and Alb proteins. Mouse kidney lysates were extracted under normoxic (N) and hypoxic (H) conditions, as indicated. The extracted kidney proteins were purified using Rab43, Pccb, or Alb antibodies, as indicated, and probed with Prdx V antibody to detect the interaction with Prdx V. [file 12953_2014_61_MOESM1_ESM.doc]
